# Trust, risk, and the challenge of information sharing during a health emergency

**DOI:** 10.1186/s12992-021-00673-9

**Published:** 2021-02-18

**Authors:** Raphael Lencucha, Shashika Bandara

**Affiliations:** 1 Faculty of Medicine, School of Physical and Occupational Therapy, McGill University, 3630 Promenade Sir William Osler, QC H3G 1Y5 Montreal, Canada; 2 Department of Family Medicine, McGill University, Montreal, Quebec Canada

**Keywords:** International Health Regulations, Health Governance, Global governance, Information sharing, Trust, Risk, World Health Organization

## Abstract

Information sharing is a critical element of an effective response to infectious disease outbreaks. The international system of coordination established through the World Health Organization via the International Health Regulations largely relies on governments to communicate timely and accurate information about health risk during an outbreak. This information supports WHO’s decision making process for declaring a public health emergency of international concern. It also aides the WHO to work with governments to coordinate efforts to contain cross-border outbreaks.

Given the importance of information sharing by governments, it is not surprising that governments that withhold or delay sharing information about outbreaks within their borders are often condemned by the international community for non-compliance with the International Health Regulations. The barriers to rapid and transparent information sharing are numerous. While governments must be held accountable for delaying or withholding information, in many cases non-compliance may be a rational response to real and perceived risks rather than a problem of technical incapacity or a lack of political commitment. Improving adherence to the International Health Regulations will require a long-term process to build trust that incorporates recognizing and mitigating the potential and perceived risks of information sharing.

## Background

One of the cornerstones of outbreak response is timely and transparent information sharing. Successful pandemic response depends on states rapidly communicating accurate information, including pathogen identification, incidence, transmission patterns, and mortality to the international community, via the World Health Organization (WHO). This information allows other states and international organizations to implement targeted and comprehensive control measures as quickly as possible. Effective communication and information sharing can also help combat the spread of ‘viral fake news’ and the ‘infodemic’ of misinformation and inaccurate and sensationalized accounts [1, 2]. Information sharing has been especially vital during the COVID-19 (SARS-CoV-2) pandemic, an easily transmissible and deadly disease which has spread in an era of ubiquitous media and communications technology.

Timely, accurate and coordinated information sharing is emphasized in the International Health Regulations (IHR), the overarching international legal agreement guiding response to health emergencies coordinated through the WHO. The IHR require governments to inform WHO of all events within their territory that may constitute a public health emergency of international concern (PHEIC) [3]. Based on the information received from a country, the WHO can declare a PHEIC and issue guidelines to control travel and trade as well as other guidance to manage the health emergency [3].

Declaring a PHEIC initiates a coordinated multi-country response to a health threat, but does not grant WHO any additional enforcement power or resources [4]. The current reporting requirements, which emerged in 2005 as a response to the limitations of the previous version of the IHR, specify that governments notify and consult with the WHO, and establish timeframes for reporting, including a 24 h window to report information that has bearing on the international spread of disease [5]. The IHR also allows WHO to collect third-party data while requiring governments to verify the information. However, WHO is currently required to report the non-governmental sources to the government, which might lead to punitive actions from governments who aim to restrict information flow [3, 6].

Unfortunately, it is common for governments to delay sharing information when facing an infectious disease outbreak. For months the governments of the three countries most effected by the 2014 Ebola outbreak consistently underreported the extent of the outbreak [7]. In 2019, Tanzania was criticized for withholding information about the Ebola outbreak within its borders [8], although the Government of Tanzania had denied these allegations [9]. During the COVID-19 pandemic, China came under intense criticism from countries like the United States and Australia for lack of transparency and delayed information sharing [10] and efforts to downplay the number of cases within its borders [11].

Conflicts over information-sharing illustrate the challenges of managing a global health emergency under the International Health Regulations (IHR). In some countries, infrastructure challenges exist driven by a lack of resources to establish proper surveillance and reporting mechanisms, and resource challenges exist within the WHO to provide technical guidance to member states [6]. In addition to surveillance, parties to the IHR are required to improve core capacities such as legislation and financing, coordination mechanisms, response to zoonotic events, food safety laboratory facilities, human resources, establishment of a health emergency framework, health service provision, risk communication, surveillance at points of entry, all of which require resources and planning. Although the WHO provides support, most countries do not have all core capacities in place [12, 13]. Although comprehensive implementation of core capacities remains a critical challenge, as of 2018 around 60–70 % of the surveillance capacity set out in the IHR has been met regionally [13].

Despite the presence of some surveillance capacity, other information challenges persist, tied in part to domestic political structures. According to Huang [14], during the SARS epidemic in 2003, China faced a delay in information flow due to bureaucratic norms such as not having personnel authorized to open reports marked “top secret,” which purportedly caused a three-day delay in transmitting information to the provincial authorities [14]. Additionally, once informed, the regional Guangzhou city government prematurely declared the disease to be under control which led to reduced public vigilance. When the government response was criticized, the provincial government halted reporting on the disease all of which contributed to the delay in informing the WHO [14]. Other extreme cases exist where a total blockage of information sharing exists. For example, Turkmekistan does not provide information about COVID-19 to international actors nor to its citizens [15]. What is more common is the ubiquitous delay in information sharing between states and the WHO.

Technical and bureaucratic challenges play an important but not determinative role in delaying information sharing. Information sharing is politically charged, and the international response to disease outbreaks has exposed a deeply entrenched politics of fear and national self-interest [16]. At the outset of the COVID-19 outbreak, countries disregarded IHR provisions by implementing blanket, unsanctioned travel bans and overly restrictive quarantine measures [17]. The implementation of these restrictions in travel come on the heels of widespread critique of similar responses to the Ebola outbreak [18, 19]. Rhymer and Speare [20] found that over 30 % of governments violated travel recommendations put forward under IHR during the Ebola outbreak. One example of such restrictions was provided by Tejpar and Hoffman [21], who scrutinized, and ultimately deemed to be a violation of the IHR, visa restrictions put in place by the Canadian government to prevent those who had visited Ebola-affected countries from entering the country.

Ultimately, the WHO relies on the voluntary participation of states to share information in a timely manner, maintain open channels of communication, and adhere to its recommendations prior to and following a declaration of a PHEIC. Nevertheless, criticism continues to be directed at the WHO’s failure to enforce state adherence to the provisions of the IHR [7, 22, 23]. WHO must weigh the benefits of declaring a PHEIC, which is vital for managing international health crises, against the costs for trade and economy. WHO defended delays in declaring a PHEIC on this basis during the 2019 outbreak of Ebola in the Democratic Republic of Congo [24, 25]. IHR champions, concerned about the risks posed to global health security when states fail to comply with their IHR commitments, urge stronger WHO leadership and enforcement. The WHO’s limited enforcement capacity coupled with the reluctance of some states to provide the international community with necessary information and resources, create ongoing challenges for managing global outbreak response.

In the remainder of this essay, we review some of the risks states face when sharing information, rooted in the recognition that trust is a foundational component of information sharing. Non-compliance may in some cases be a rational response to real and perceived risks rather than a lack of technical competence or political commitment. IHR implementation thus requires trust and mutual recognition of both the benefits and risks of information sharing for states [16]. These risks occur within a global context in which inter-state relations are marked by power imbalances and legacies of mistrust, which have been exacerbated during the COVID-19 pandemic. Following the COVID-19 pandemic the WHO will likely coordinate revisions to the IHR [26]. This revision should address the long-standing challenges related to information sharing under the IHR, including the political and economic concerns of member states. To begin, we outline the detrimental consequences of reporting.

### The consequences of reporting

The economic impacts of infectious disease pandemics are well documented. States that report information about disease outbreaks risk considerable economic losses stemming from formal restrictions on travel and trade by states, as well as voluntary restrictions on consumption or travel by individuals and private entities based on perceived risk. For example, during the SARS outbreak hotels in China lost 80 % of their guests. The number of visitors to common tourist attractions in Bejing also fell by 80 %, and revenue dropped rapidly and dramatically for restaurants and domestic transport [27]. Researchers cite WHO travel restrictions as one of the reasons that caused this reduction in tourism, although other factors may have contributed to this reduction as well [28]. Other analyses found that total tourist arrivals decreased by 30 % in April and May of 2003 following the SARS outbreaks [28], and that SARS cost the global economy an estimated US $ 40 billion [29, 30] Preliminary analyses suggest that the economic consequences of the COVID-19 pandemic, which has proven much more difficult to contain than SARS, are likely to be substantially higher [31, 32].

Reporting can have regional consequences as well, even among countries unaffected by an outbreak. A report from the World Travel and Tourism Council published in 2018 found that the entire continent of Africa saw a precipitous decline in tourism after the 2016 Ebola outbreak became publicized globally [33]. Despite extremely low risk, countries like Kenya that were geographically distant from the outbreak, reported declines in visitors with the expressed reason being fear of contracting Ebola. Studies conducted following the Zika [34], H1N1 [35, 36], and Avian Influenza [37] outbreaks found similar impacts on regional tourism. Countries may thus face external pressure to downplay reporting from neighboring states heavily dependent on tourism.

Countries also suffer economic consequences resulting from trade restrictions and withdrawal of investment in local industries. Following the 2009 H1N1 outbreak, Mexico saw massive decreases in the export of chilled and fresh pork, over a 60 % reduction to Japan [38], and an overall pork trade deficit of US$27 million by the end of 2009 [35]. While in some cases restrictions may be justified, in others they are driven more by perceived than actual risk [35]. In either case, the economic impact is real. While the IHR provisions provide a moderate and balanced approach to containment, countries consistently implement unreasonable restrictions on travel and trade [39]. Despite the hope that the WHO’s neutral stance and expertise would be useful in limiting unnecessary restrictions on travel and trade [40], both during the 2014 and 2019 Ebola outbreaks and early in the COVID-19 outbreak, governments blatantly disregarded the WHO and implemented restrictions beyond what the organization had recommended [21, 41].

Information-sharing occurs within a context shaped by the historical legacy of colonialism, as well as persistent inequalities driven by extractive relationships between wealthy states and private enterprises largely based in the global north and resource-rich states in the global south. This dynamic contributes to a deep lack of trust among nations that are expected to share information that could be economically damaging to themselves, while benefiting wealthier countries’ security and economic interests. Indeed, some suggest that the IHR is advantageous for wealthier countries, who can quickly mobilize resources to combat novel pandemics. By contrast, poorer countries may have to divert limited public health financial and human resources from endemic public health threats such as HIV, tuberculosis and malaria, simply in order to comply with IHR regulations [6].

This lack of trust tied to an economic imbalance between states is illustrated by a controversy around virus sharing beginning in 2006. After the H5N1 outbreak, Indonesia shared virus samples with the WHO affiliated laboratories under the Global Influenza Surveillance Network (GISN) [42]. It was later determined that pharmaceutical companies were patenting modified strains of the virus collected from Indonesia [43]. Upon discovering that these samples had been shared outside the GISN without the country’s consent, Indonesia stopped sharing samples on the grounds of state sovereignty, the grundnorm of international relations [44], and the need to ensure that Indonesian citizens benefit from vaccine development. In response, the United States delegation to the World Health Assembly argued that “all nations have a responsibility under the revised IHRs to share data and virus samples on a timely basis and without preconditions … withholding influenza viruses … greatly threatens global public health and will violate the legal obligations we have all agreed to undertake through our adherence to IHRs” [45]. The WHO subsequently introduced the Pandemic Influenza Preparedness Framework in 2011, which intended to secure access to influenza viruses and assured fairer distribution of vaccines and other benefits associated with the use of samples [46].

Finally, mistrust is sustained by racism, discrimination, and the continued use of xenophobic rhetoric in the context of pandemic response. American media, echoing a broader anti-immigrant discourse, frequently portrayed H1N1 as ‘Mexico’s swine flu’ [47] (p. 728). A former interior minister of Italy incorrectly blamed African asylum seekers for the spread of COVID-19, and called for restrictions on immigration from Africa [48]. US President Donald Trump had referred to COVID-19 as the “Chinese virus,” linking it to broader anti-China sentiments regarding trade [49].

### Navigating tensions

The tendency to focus on the technical aspects of IHR implementation and enforcement have the potential to obscure the deep socio-political foundations of international cooperation and coordination [7, 50, 51]. The persistent, perhaps perennial, challenge is how to encourage states to move towards cooperation and coordination. When states legitimately fear that sharing information will have negative political and economic consequences, they are disincentivized from complying with IHR guidelines. We agree with Sara Davies’ assertion that “what is needed is more communication, more information, and more efforts to build trust between states, the WHO, and the citizenry” [50], and believe that trust is a necessary pre-requisite for information-sharing. The first step towards building trust is recognizing and addressing the disincentives to information sharing.

Layered into the problem of overly restrictive or ‘illegal’ reactions by states on travel to and trade with ‘origin’ or high-risk countries is the deliberate withholding of information from the international community by governments. The tension between China and those who accuse its government of delaying the sharing of information at the outset of the COVID-19 outbreak and continuing to withhold important information from the WHO and other countries illustrates how quickly conflict can escalate between states during a pandemic. The obfuscation coming from multiple sides suggests that it might be some time before we learn whether and to what extent China deliberately withheld information. We are already seeing analyses that seem to exonerate China [52], while others criticize countries like the United States for obscuring information distributed to the public [53]. However, we do know that the practice of concealing information is common, with major implications for how governments and intergovernmental agencies are able to coordinate a response [54]. The fact that information sharing entails risks does not justify the deliberate and nefarious withholding of information; but it does help illuminate some countries’ reticence to share, in some situations.

The reasons for delayed or incomplete information sharing may differ depending on the situation. Consider, for example, the case of reporting by Guinea during the Ebola outbreak. In this case, the scale and nature of the outbreak posed challenges to collecting accurate information, moving it through formal channels of government, and eventually sharing it with the WHO [7]. Governments first need to have the information infrastructure to monitor, evaluate and transmit information through the necessary channels. We know that this remains a structural barrier to coordination, given that the majority of IHR member states have not implemented the core capacities [55, 56]. Nevertheless, perceived domestic and international political risks in sharing the extent of the outbreak were also a factor, and there were economic consequences to tourism and trade when the information was shared.

One possible way to address information sharing problems is through the development of complementary information systems that function in parallel to government reporting mechanisms. For example, the Global Outbreak and Response Network (GOARN), including organizations such as ProMED which is a non-profit organization that was one of the first to indicate that an outbreak was emerging in Wuhan, is a network of technical agencies and organizations, coordinates support for preparedness and response, and facilitates surveillance infrastructure and training, but is not a formal information channel [57]. Similarly, the Global Public Health Intelligence Network surveys media sources for indications of outbreaks, and issued the first alert of the SARS outbreak in the Guangdong Province of China [58]. Non-state actors may also take on the role of information sharing. During the Ebola outbreak in Guinea, Médecins Sans Frontières played an important role in sharing information with the international community [59]. However, reliance on non-traditional sources of information poses its own problems regarding the validity and reliability of data and data sources [59]. Moreover, while complementary systems may improve outbreak detection and response, they do not directly address the underlying political and economic concerns that may lead to non-compliance in the first place. Despite this tenuous and delicate political context, we are already seeing non-state actors taking on the role of information sharing, which was the case during the Ebola outbreak in Guinea when Médecins Sans Frontières played an important role in sharing information with the international community [59].

The expanded range of information sources will create “a new set of coordination challenges emerge in terms of the validity and reliability of data and data sources” [59]. Such challenges are already being addressed in other domains of information sharing, and lessons can be drawn from these initiatives. For example, the surveillance system for attacks on healthcare (SSA) facilities and workers categorizes information based on level of confidence as “rumor”, “possible”, “probable” and “confirmed” [60]. Having a typology to verify the trustworthiness of the information source will be important. In an attempt to coordinate information sharing across state and non-state actors who collect public health intelligence, such as disease outbreak information, WHO initiated the Epidemic Intelligence from Open Sources (EIOS) in 2017 [61]. The main goal of EIOS is to facilitate early outbreak detection by consolidating public health intelligence from all open sources and to build a web-based system that streamlines information sharing. By creating this web-based platform, WHO aims to reinforce surveillance of health risks. EIOS is led by a twelve member coordination body that includes United Nations organizations, ministries of health, and public health agencies for two year terms [61]. So far EIOS has been used to identify rumor patterns related to Zika, early detection of African Swine Flu, and currently is collating through COVID-19 news to provide a news map on COVID-19 [61, 62]. One limitation that EIOS has faced is the country level control of information which can hamper the availability, accessibility and timeliness of information.

Ultimately, compliance requires trust, which in turn depends upon improved transparency [6, 63]. Mechanisms for increasing transparency include ensuring that the WHO follows explicit and objective criteria when declaring a PHEIC and providing countries with decision making tools or a methodology to assess the appropriate level of trade and travel restrictions. Yet transparency alone is unlikely to sufficiently incentivize greater information-sharing if the brunt of negative consequences is disproportionately borne by poorer countries. When revising the IHR, the WHO should consider implementing mechanisms to mitigate extant political and economic inequalities and by, for example, establishing a dedicated fund to support countries who adhere to reporting guidelines and may be most impacted by restrictions. Existing funding structures such as World Bank’s Pandemic Emergency Financing Facility (PEF) aim to provide support for International Development Association member countries when facing cross border outbreak threats [64]. During COVID-19, the main weaknesses of PEF has been the delayed release of funds and having what some consider overly stringent criteria for fund release [65]. Identifying, learning and addressing weaknesses in existing mechanisms such as PEF or building more robust funding mechanisms with higher capacity will be crucial moving forward [66]. Furthermore, mechanisms and policies are needed to ensure equitable distribution of medical solutions such as vaccines, therapeutics and diagnostics that are crucial to responding to a PHEIC [67]. The WHO launched the Access to COVID-19 Tools (ACT) Accelerator in April 2020 as a mechanism to coordinate “to accelerate development, production and equitable access to COVID-19 tests, treatments and vaccines” [68]. Like the WHO itself, the initiative remains seriously underfunded and has been unable to curb the nationalistic orientation of governments in their approach to vaccine acquisition and distribution, leaving advocates of the program appealing to return-on-investment for countries willing to support an equitable global approach [69]. The example of ‘vaccine nationalism’ is emblematic of the deep and persistent issues of international relations that fuel mistrust between states, further entrenching a disjointed and ad hoc response to health emergencies [70].

An emphasis on trust-building does not lend itself to clear, simple or short-term solutions. However, it does offer a more comprehensive assessment of the challenges to international cooperation that extends beyond the realm of the technical or the procedural. As Brown and Ladwig [71] pointedly note, what choice does the WHO have but to appease states who do not follow the rules, when governments continue to withdraw funding international agencies [72] and hollow out the authority of the WHO in international agreements [73]. Additionally, what are states to do when the consequences of sharing outweigh the benefits. One of the persistent challenges to trust-building is the ‘border orientation’ of governments [74]. This orientation externalizes health threats to the ‘other’ rather than embracing domestic control measures, focusing on who needs to be kept out rather than investing in a comprehensive and sustained global approach to containment. This impulse to externalize and politicize health threats runs contrary to effective approaches to response that involve coordination and cooperation [75].

## Conclusions

It is widely accepted that withholding information is a pivotal and perennial challenge for rapid and coordinated response. Equally apparent is the corrosive impact of overly restrictive, xenophobic and racist responses to outbreaks. Facilitating a better response to pandemics will require both the WHO and member states to implement trust-building measures, including better transparency and formal mechanisms to address the negative impacts of information sharing. The challenge of trust-building is not easy but warrants attention if the system of outbreak response is to improve.

## Data Availability

Not applicable.

## Abbreviations

WHO: World Health Organization
IHR: International Health Regulations
PHEIC: Public Health Emergency of International Concern
GOARN: Global Outbreak and Response Network
GISN: Global Influenza Surveillance Network
EIOS: Epidemic Intelligence from Open Sources
SSA: Surveillance System for Attacks on Healthcare
PEF: Pandemic Emergency Financing Facility
